# Development of sensitive and specific immunochromatographic strips with nanoparticles for rapid detection of flumequine

**DOI:** 10.1016/j.fochx.2025.102771

**Published:** 2025-07-09

**Authors:** Yuxuan Liu, Kang Jiang, Xiaoli Li, Linfang Lu, Menghan Sun, Na Li, Lakshani Madushika, Jun Yuan, Sumei Ling, Shihua Wang

**Affiliations:** a The Ministry of Education Key Laboratory of Biopesticide and Chemical Biology, Fujian Agriculture and Forestry University, Fuzhou 350002, China; b Fujian Key Laboratory of Pathogenic Fungi and Mycotoxins, Fujian Agriculture and Forestry University, Fuzhou 350002, China; c College of Life Sciences, Fujian Agriculture and Forestry University, Fuzhou 350002, China

**Keywords:** Flumequine, Monoclonal antibodies, Nanoparticles, Immunochromatographic strips

## Abstract

Flumequine (Flu) is a fluoroquinolone veterinary antibiotic, which is easy to accumulate in animals, and Flu residues may cause a latent risk to human physiological health. In this study, a high-affinity monoclonal antibody (2.09 × 10^9^ M^−1^) to specifically recognize Flu (anti-Flu mAb) was prepared, and then the lateral flow immunochromatographic strips (LFIS) with excellent sensitivity and specificity were developed by combining mAbs with nanoparticles. The linear detection ranges of LFIS based on gold particles (AuNP-LFIS) and gold nanoflowers (AuNF-LFIS) were 1.95–250 ng/mL and 0.39–100 ng/mL, respectively, and two types of LFIS showed high sensitivity and accuracy in actual sample detection. It is noteworthy that the AuNF-LFIS exhibited a higher sensitivity compared to that of the AuNP-LFIS. The LFIS developed in this study were considered to have great application potential in monitoring food safety because of its superiorities including simple operation, high sensitivity, prompted speed and good specificity.

## Highlights

• Anti-Flu monoclonal antibody with high affinity (2.09 × 10^9^ M^−1^) was obtained.
• Gold nanoparticle (AuNP) and Gold nanoflower (AuNF) were prepared as signal reporters.
• Sensitivity of immunochromatography was enhanced using AuNF nanoparticles.

## 1 Introduction

Following the progress of animal husbandry and breeding industry, veterinary drugs have gradually become an indispensable part of the breeding process (Khadim et al., 2023; Suryawanshi et al., 2024). Flumequine (Flu) is a fluoroquinolone antibiotic, which can inhibit the synthesis of bacterial nucleic acids by targeting bacterial DNA gyrase and topoisomerase (Higgins, Fluit, & Schmitz, 2003). As a special antibacterial drug for animals, Flu is widely used to treat diseases in various food animals, such as poultry, livestock, and aquaculture. Taking the increased bacterial resistance and risks of cancer induction, the widespread use and abuse of flumequine may cause latent risks to human health (Anadón et al., 2008; Kang et al., 2018). For the sake of protecting the food safety of the public, lots of countries have expressly stipulated the maximum residue limits (MRLs) of Flu in food. For example, in Chinese national standard GB31650-2019 and GB-31650.1-2022, the MRLs of Flu in meat, egg, and milk are 500 μg/kg, 10 μg/kg, and 50 μg/kg, respectively. In summary, it is essential to establish a handy and sensitive, specific and speedy method for detecting Flu.

Over the past few decades, a variety of different liquid chromatography (Karbiwnyk, Carr, Turnipseed, Andersen, & Miller, 2007; Tayeb-Cherif, Peris-Vicente, Carda-Broch, & Esteve-Romero, 2016) and enzyme immunoassay (ELISA) methods (Hendrickson, Zherdev, & Dzantiev, 2024; Van Coillie, De Block, & Reybroeck, 2004) had been widely used for detecting Flu in food animal samples. Liquid chromatography is known for its precise analytical results and minimal error (Chen et al., 2022; Gong, Chen, Zhang, Li, & Song, 2023; Jingshun Zhang et al., 2022). However, liquid chromatography often requires lengthy analysis time, complex pre-processing, and expensive equipment support. Compared to liquid chromatography, enzyme immunoassay has shorter detection time, easier sample pretreatment and lower cost (Vu et al., 2023). Nevertheless, for the detection of plenty of samples, the procedure of enzyme immunoassay is still cumbersome. Thus, there is a highly urgent need for a sensitive and specific method with faster detection speed to monitor Flu residues in large quantities of animal-sourced products.

In recent years, lateral flow immunochromatographic strips (LFIS) were broadly applied to the qualitative or quantitative detection as a hypersensitive and highly specific detection method with low cost, simple operation and short analysis time (Gupta & Ghrera, 2021). LFIS were based on antibodies that specifically recognize the substance to be detected and various markers that label antibodies (Ma, Guo, Jiang, & Zhao, 2024). Among these antibody markers, gold nanoparticle (AuNP), as the most typical nanomaterial, was widely used in various immunochromatography methods due to its excellent biocompatibility, ease of synthesis and discernible color properties (Ling et al., 2022; Jiangjiang Zhang, Chai, Li, et al., 2024; Zhang, Mazouzi, Salmain, Liedberg, & Boujday, 2020). Although the LFIS utilizing the traditional AuNP nanoparticle exhibited the advantage of rapid and high selective to detect target analysts, there are still challenges in sensitivity owing to the low optical signal intensity and limited surface area for antibody immobilization (Zhu et al., 2008). To address these challenges, gold nanoflower (AuNF) with large surface area and strong optical signal was considered to be ideal candidate to enhance the immunochromatographic sensitivity (Huang et al., 2025; Ling et al., 2024; Zuo, Yan, Tang, Zhang, & Li, 2023). To date, there is no a LFIS method for specific detection of Flu based on anti-Flu monoclonal antibodies (mAbs) till now.

**Scheme 1:**
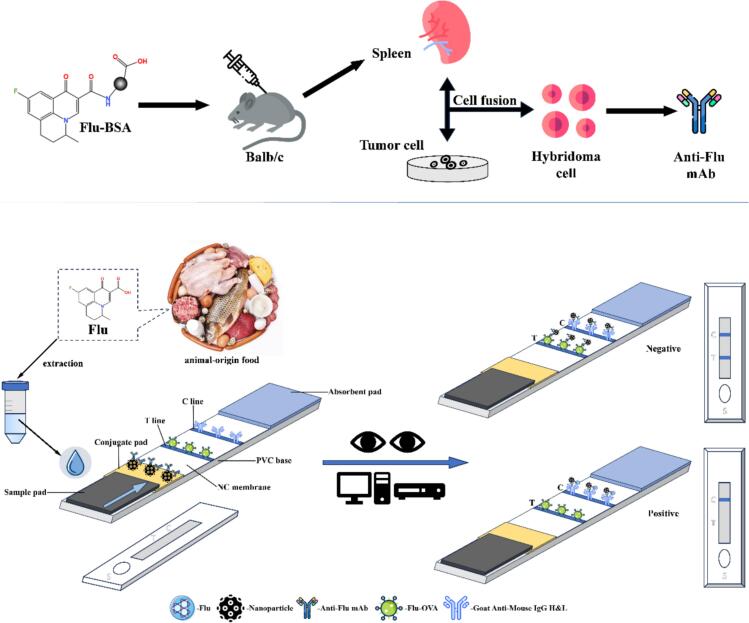
Schematic illustration of the anti-Flu mAb preparation and LFIS method for Flu detection. (A) The anti-Flu mAbs preparation process starting from injection of complete antigens. (B) The detection process and working principle of this LFIS.

In this study, a sensitive and specific LFIS based on AuNP and AuNF probes to amplify the signal was developed for detecting Flu (Scheme 1). At first, the complete antigen Flu-BSA and Flu-OVA were synthesized as immunogen and test antigen respectively. Then, a high affinity anti-Flu monoclonal antibody (mAb) was obtained after animal immunization, cell fusion, ascites induction and antibody purification. In addition, AuNP and AuNF were synthesized and combined with antibodies to form complete probes. Moreover, strips were assembled based on the probes and evaluated by establishing standard curves, detection limits, and specificity. Finally, we detected actual samples using our established strips and compared strip method with the liquid chromatography-mass spectrometry (LC-MS) method.

## 2 Materials and methods

### 2.1 Reagents and experimental animals

Analytical standard Flu, Albumin from chicken egg white (OVA), Bovine serum albumin (BSA), Freund's incomplete adjuvant, and Freund's complete adjuvant were purchased from Sigma-Aldrich (Saint Louis, USA). Standards for enrofloxacin (Enr), ciprofloxacin (Cip), levofloxacin (Lof), marbofloxacin (Mar), norfloxacin (Nor), and *N, N*-dimethylformamide (DMF) were purchased from Aladdin Biochemical Technology (Shanghai, China). N-Hydroxy succinimide (NHS) and 1-(3-Dimethylaminopropyl)-3-ethylcarbodiimide (EDC) were from TCI (Shanghai) Development Co., Ltd. (Shanghai, China). Fetal Bovine Serum was from Sangon Biotech (Shanghai, China). Protein G resin was from GenScript (Nanjing, China). Gold (III) chloride trihydrate (HAuCl_4_•3H_2_O) and Hydroquinone were obtained from Macklin Inc. (Shanghai, China). Goat anti-mouse IgG (Gam IgG) was from Bioss Biotech (Beijing, China).

6-week-old and 10-week-old female Balb/c mice (from SLACCAS (Shanghai, China)) were kept in a well-fed, clean and comfortable living environment during the experiment. At the end of the study, all of Balb/c mice were euthanized by cervical dislocation after inhaling isoflurane. The dead mice were collected and disposed of in bio-waste bags (Dong, Qin, Li, Wu, & Liu, 2022; Liu et al., 2020). We advocated minimizing animal experiments as much as possible. All animal experiments were approved by the Ethics Committee (license number: PZCASFAFU24118, date: 6 June 2024) of the Fujian Agriculture and Forestry University of China.

### 2.2 Synthesis and identification of complete antigens

The complete antigens were synthesized by carbodiimide method, and the proportion of components in the reaction system was adjusted according to previous studies (Han et al., 2023; López-Puertollano, Agulló, Mercader, Abad-Somovilla, & Abad-Fuentes, 2020; Yin et al., 2023). Firstly, 4.18 mg Flu, 5.52 mg *N*-Hydroxy succinimide and 8.48 μL 1-(3-Dimethylaminopropyl)-3-ethylcarbodiimide were dissolved in 0.4 mL DMF (the molar ratios of the reaction system: Flu:NHS:EDC = 1:3:3) and reacted in a constant temperature shaker at 20 °C, 110 r/min for 12 h. Next, the fully reacted system in the previous step was added drop by drop to 3.6 mL of 5 mg/mL BSA/OVA solution (in 0.01 M PBS, pH 8.5) and reacted fully in the shaker at 25 °C, 110 r/min for 6 h. Then, the reaction fluid was dialyzed at 4 °C in 0.01 M PBS (pH 7.4) for three days. Finally, the complete antigen Flu-BSA/OVA was obtained, verified by agarose gel electrophoresis, and confirmed by ultraviolet spectrophotometer.

### 2.3 Preparation of hybridoma cell line

Flu-BSA was selected as the immunogen, diluted to a suitable concentration with 0.01 M PBS, and then emulsified with equal volume of complete adjuvant. 6-week-old Balb/c female mice with an average weight of 20 g were selected and injected with the fully emulsified emulsion. Thenceforward the same batches of mice were immunized every two weeks with the emulsified emulsion of the same immunogen and incomplete adjuvant. After the second immunization, Flu-OVA was used as the coating antigen to determine the antibody titer and specificity of the serum from the mouse tail vein by indirect ELISA. On the fourth day after the fourth immunization, the mice with the highest serum antibody titer and the best specificity were subjected to shock immunization with 0.01 M PBS containing 100 μg immunogen. On the next day, the mouse's spleen was removed and spleen cells were obtained. PEG 1450 was then used to mediate the fusion of spleen cells with SP2/0 mouse myeloma cells. After subcloning and screening in cell culture, a stable hybridoma cell line with anti-Flu mAbs was obtained. The subtypes and specificity of antibodies secreted by the cell line were identified by kit (from Proteintech Group, Inc.) and indirect competitive ELISA (icELISA), respectively (Cai, Wang, Ling, & Wang, 2021).

### 2.4 Acquisition and characterization of anti-flu mAbs

An appropriate amount of stable antibody-secreting hybridoma cells obtained in 2.3 were injected intraperitoneally into 10-week-old Balb/c female mice. Approximately one week later, the ascitic fluid from the mouse was collected. Then, the anti-Flu mAb was purified from the ascitic fluid using Protein G method. The antibody purification details showed as follows. After centrifugation, the supernatant was subjected to a Protein G affinity column that had been pre-equilibrated. The target antibodies were eluted using a low-pH glycine-HCl buffer (0.1 M, pH 2.7) and dialyzed at 4 °C in 0.01 M PBS (pH 7.4) for three days (Ling et al., 2015).The purified antibodies were identified by SDS-PAGE. The affinities of the mAbs produced by different cell lines were then determined by ELISA, and the calculation method for affinity is referred to a previous study (Tan et al., 2023). After that, the sensitivity and specificity of mAbs with higher affinity were determined by icELISA. Subsequently, the best-performing antibodies would be used to make probes.

### 2.5 Preparation and pre-treatment of AuNP probes

AuNPs were fabricated with reference to the previous method (Tang et al., 2022). Above all, 1 mL aqueous solution of 1 % HAuCl_4_ was added into a clean flask filled with 100 mL deionized water that was heating, and then 2 mL of 1 % trisodium citrate solution was added when the liquid was nearly completely boiling. The conjugation of AuNP probes was carried out in accordance with precedents established in previous research (Byzova, Safenkova, Slutskaya, Zherdev, & Dzantiev, 2017; Geng et al., 2016) and the coupling condition of anti-Flu mAbs to AuNPs was determined by antibody protein flocculation test.

### 2.6 Preparation of AuNFs and AuNF probes

AuNFs were fabricated on the basis of a previous study (Ji et al., 2015) with some modifications. First, a batch of AuNPs were prepared in a similar way as in 2.5 (except that the addition of 1 % trisodium citrate in the preparation process was changed to 1.5 mL). In addition, 750 μL aqueous solution of 1 % HAuCl_4_ was added to a clean flask filled with 100 mL deionized water. With stirring slowly and continuously at room temperature, solutions of 500 μL AuNP and 300 μL 1 % trisodium citrate was added successively to the flask. Next, the pH was adjusted using 1 M NaOH. Finally, 1 mL of 0.03 M hydroquinone solution was added to the flask and the stirring speed was increased until the solution color turned a clear dark blue for 5 min.

Then, the synthesized AuNFs was used to conjugate with anti-Flu mAb for the formation of AuNF probe. Firstly, the optimal pH solution and appropriate amount of anti-Flu mAbs were determined. The AuNF probe was synthesized under the optimal conditions. Appropriate anti-Flu mAbs were added to a certain amount of AuNF solution with slowly stirring in ice bath, and continued stirring for 45 min. Next, 5 % BSA aqueous solution was added to the coupled system and stirred at low speed for 25 min. Following this, 1 % PEG20000 aqueous solution was added and stirred at low speed for additional 30 min. After centrifugation at 12,000 r/min for 40 min, the obtained of AuNF probes were re-suspended in a 20 μL mixture solution (0.01 M Tris-HCl + 1 % BSA + 10 % trehalose, pH 8.5). The obtained probes were stored at 4 °C for later use.

### 2.7 Construction and principle of LFIS

Our LFIS were assembled with reference to our previous research, which included NC membrane, absorbent pad, conjugate pad, sample pad and PVC base (Ling et al., 2021). Goat anti-mouse IgG and Flu-OVA were respectively coated on the C line and T line of the NC membrane with PVC base, with a separation of 6 mm between these two lines. After that, the NC membrane with PVC base was dried at 37 °C for 3 h. Subsequently, absorbent pads, conjugate pads and sample pads were pasted on strips. Among them, conjugate pads had been bound with the treated probes that dried at 37 °C for 30 min. Based on the above LFIS assembly method, the optimal working conditions of LFIS were determined by adjusting the additive amount of Goat anti-mouse IgG, Flu-OVA and probes in strips.

The princible of the developed LFIS was described as follows. When a negative sample was detected, the probes on the conjugate pad flowed to the absorbent pad with the drip of the sample liquid. Anti-Flu mAbs in probes bound to Flu-OVA on the T line until T line is saturated, then remaining probes continued to flow to the C line, and the anti-Flu mAbs in probes bound to the goat anti-mouse IgG on C line. Finally, the results showed that the color of T line and C line were clearly visible. When a positive sample was detected, Flu flowed to the absorbent pad with the drip of the sample liquid. Flu bound with anti-Flu mAbs in the probes when it passed through conjugate pad, causing probes to flow directly to the C line instead of binding to Flu-OVA on the T line, and then anti-Flu mAbs in probes bound to the goat anti-mouse IgG on the C line. Finally, the result indicated that T line showed no color while C line showed color. The entire reaction of our LFIS took about 10 min.

### 2.8 Evaluation of LFIS

Sensitivity and specificity were two important indicators to evaluate LFIS performance. To assess the sensitivity of the LFIS performance, a range of Flu concentrations ranging from 2000 ng/mL to 1.95 ng/mL were examined, with PBS serving as the negative control. The limit of detection (LOD) was defined as the target concentration (IC_10_) corresponding to 10 % inhibition of the value in many previous studies (Hua et al., 2017; Huang et al., 2023). In this study, to reduce the errors caused by standard curve fitting, LOD was considered as the Flu concentration corresponding to the point whose horizontal coordinate value was closest to IC_10_ and higher than IC_10_ in the standard curve. The signal value was represented by B/B_0_, which was defined as the ratio of I_T_/I_C_ (the ratio of the color intensity of T line to the color intensity of C line) value of positive (with Flu) to negative (without Flu). The color intensity was recorded with an immunochromato reader (HAMAMATSU, Hamamatsu, Japan). In addition, since the results of our LFIS can be directly observed with the naked eye, we also defined the visual limit of detection (vLOD) that was the Flu concentration when the color of the T line was just clearly lighter than that of the C line under the naked eye. The specificity of our AuNP-LFIS and AuNF-LFIS was evaluated employing structurally analogous fluoroquinolones, including Mar, Nor, Cip, Lof, and Enr. To more accurately reflect real-world cross-reactivity risks, we have assessed the specificity of the proposed LFIS by examining groups of Flu at high, medium, and low concentrations (1 μg/mL, 0.2 μg/mL, and 0.04 μg/mL for AuNP-LFIS, while 200 ng/mL, 40 ng/mL and 8 ng/mL for AuNF-LFIS). These structurally analogous compounds were diluted to 1 ng/mL, then subjected to testing for AuNP-LFIS, whereas 200 ng/mL was for AuNF-LFIS (Zhang et al., 2022; Chen et al., 2022). All the tests were conducted in triplicate.

### 2.9 Detection of samples

Pork, fish, milk, and egg samples were randomly purchased from Yonghui Superstores (Fujian, China). The pre-treatment method of samples was improved according to GB/T 21312-2007 (China). To be specific, 5 g homogeneous sample and 10 mL 0.1 M EDTA-McIlvaine buffer were added into a 50 mL centrifuge tube, low-speed vortex extraction for 1 min and ultrasonic extraction for 10 min. After the initial extraction, 10 mL of 0.1 M EDTA-McIlvaine buffer was added and the high-speed vortex extraction was carried out for 3 min. After centrifuging at 4000*g* for 10 min, 1 M NaOH was used to adjust the pH of the supernatant to neutral. Next, the neutral supernatant was centrifuged at 2000*g* for 10 min, and the new supernatant was purified with 0.45 μm filter membrane. The extractive solution was then used for testing and spiking.

Fish and milk samples were used as spiked samples. For AuNP-LFIS, the recoveries of spiked samples were calculated with Flu at spiked concentrations of 10, 40, and 160 μg/kg. For AuNF-LFIS, the recoveries of spiked samples were calculated with Flu at spiked concentrations of 5, 20, and 80 μg/kg. The reliability of spiked recovery had been verified by LC-MS (Sun et al., 2022).

### 2.10 Data analytics

The ELISA tests for characterizing antibodies were determined with three triples. The immunochromatographic results of our strips were repeated with three triples. All experimental data were recorded and calculated by Microsoft Excel 2019. The drawing of standard curves and linear analysis were performed by Origin 2021. Bar, line and curve charts were drawn by GraphPad Prism 8.3.0.

## 3 Results and discussion

### 3.1 Verification of complete antigens

**Fig. 1:**
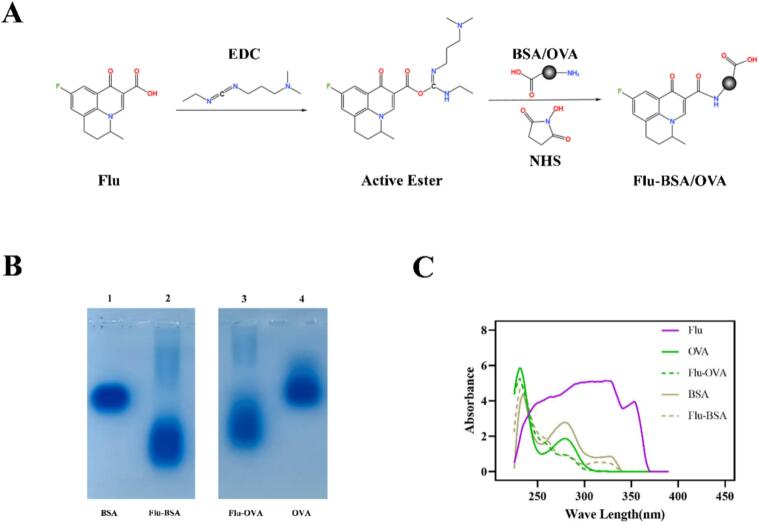
Synthesis and verification of Flu complete antigens. (A) Synthesis process of Flu complete antigens. (B) Analysis of Flu-protein conjugates using agarose gel electrophoresis (lane 1: BSA, lane 2: Flu-BSA, lane 3: Flu-OVA, lane 4: OVA). (C) UV–vis analysis of Flu complete antigens.

Flu has a simple chemical structure with a molecular weight of only 261.25 and is not immunogenic by itself. In view of the structural characteristics of Flu, the carbodiimide method was used to link the carboxyl group position of Flu with the amino group position on the surface of the carrier protein (Fig. 1A). Fig. 1B showed the agarose gel electrophoresis results of Flu-BSA and Flu-OVA synthesized by the same method. Because the proteins in the complete antigen were bound by Flu, the complete antigen carried more charge than the original carrier protein. Thus, the migration rate of Flu-BSA/OVA in agarose gel was higher than that of BSA/OVA. As shown in Fig. 1C, the absorption peaks of the complete antigens at around 280 nm were shifted compared with the corresponding carrier proteins, and their waveforms observably changed as well. The above results proved that the conjugation of complete antigen was successful.

### 3.2 Preparation and characterization of anti-flu mAbs

**Fig. 2:**
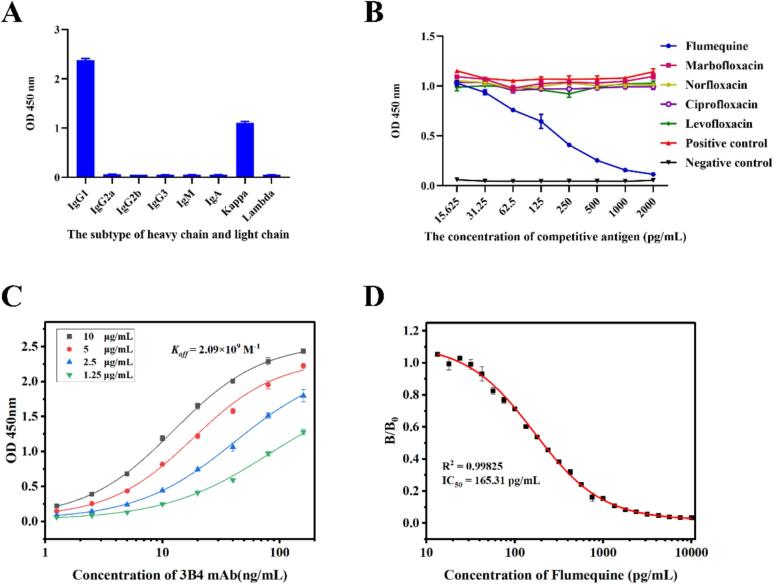
Identification and evaluation of anti-Flu mAb performance. (A) Subtypes of antibodies secreted by 3B4 cell line. (B) Specificity of antibodies secreted by 3B4 cell line by icELISA. (C) Affinity analysis of anti-Flu mAbs purified from ascites induced by 3B4 cell line. (D) The competitive inhibition curve of icELISA for detecting Flu.

In the process of animal immunity, different mice had different immune responses to complete antigens. The individual with the best immune response was selected by evaluating antibodies in the serum of mice. In the same batch, the stronger the specific recognition ability of antiserum to Flu, the better the corresponding immune effect of mice (Ling et al., 2020). After the fusion of spleen cells from the selected individual with SP2/0 cells, a cell line 3B4 with strong activity and high specificity of antibody recognition was obtained after screening. The subtypes and specificities of the antibodies secreted by this cell line were shown in Fig. 2A–B. Anti-Flu mAbs purified from ascites induced by 3B4 cell line have high affinity, and the affinity constant (*K*_*aff*_) of the antibody-antigen reaction was 2.09 × 10^9^ M^−1^ (Fig. 2C). The IC_50_ of icELISA established under optimal antigen-antibody response conditions were 165.31 pg/mL (Fig. 2D). The high sensitivity of ELISA laid an excellent foundation for the subsequent development of LFIS.

### 3.3 Characterization of AuNPs/AuNFs and AuNP/AuNF probes

**Fig. 3:**
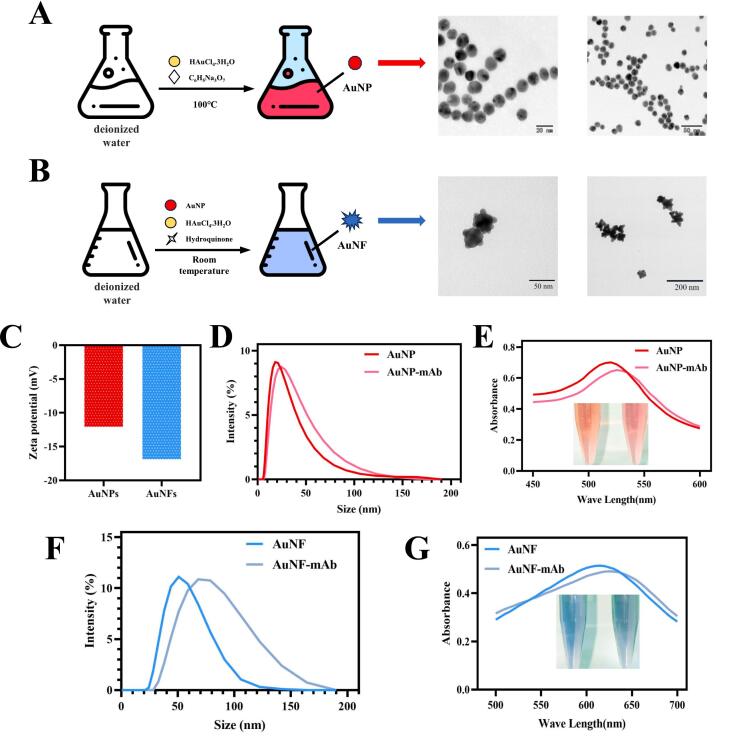
Characterization of nanoparticles and probes. (A) Diagram and TEM images of AuNPs. (B) Diagram and TEM images of AuNFs. (C) Comparison of zeta potential between AuNP and AuNF. (D) Particle size distributions of AuNP and AuNP-mAb by DLS analysis. (E) The changes of color and UV–vis spectrum between AuNP and AuNP-mAb. (F) Particle size distributions of AuNF and AuNF-mAb by DLS analysis. (G) The changes of color and UV–vis spectrum between AuNF and AuNF-mAb.

The shape and size of nanoparticles have prominent influence on their function and stability. The AuNPs were synthesized via the reduction of chloroauric acid employing sodium citrate as the reducing agent and the resulting particles exhibited a spherical morphology with an average diameter of approximately 20 nm (Fig. 3A). The AuNFs were obtained through a seed-mediated growth method utilizing the synthesized AuNPs as the gold seeds and the synthesized AuNFs were multi-branched structure with a diameter of about 50 nm (Fig. 3B). In addition, the synthesized AuNPs and AuNFs had high zeta potential values (−12.1 ± 1.1 and − 16.9 ± 0.6), which indicated that they had good stability (Fig. 3C).

Then, the prepared AuNPs were used to couple with anti-Flu mAb for the formation of AuNPs under the optimal coupling conditions with the 0.75 μL of 0.1 M K_2_CO_3_ and 3 μL of anti-Flu mAbs (0.4 mg/mL). As depicted in Fig. 3D, after conjugation with mAb, the average sizes of AuNP-mAb were larger than those of AuNP particles. The UV–vis analysis showed that λ_max_ absorbances of AuNP and AuNP-mAb were 520 nm and 525 nm, respectively (Fig. 3E). The above results the AuNP-mAbs were successfully obtained. Furthermore, the AuNF probes were synthesized under optimal conditions utilizing 0.5 μL of 0.1 M K_2_CO_3_ and 2 μL of anti-Flu mAbs (0.4 mg/mL). The average sizes of AuNF-mAb were larger than those of AuNF particles (Fig. 3F). The successful conjugation of anti-Flu mAb onto the surface of AuNFs was further confirmed by UV–vis spectrum, where the λmax of AuNF-mAb shifted from 615 nm to 625 (Fig. 3G). These findings indicated that both AuNP and AuNF probes were successfully synthesized and could be used as signal reporters in the development of LFIS.

### 3.4 Development and evaluation of LFIS

The proposed LFIS was assembled with the AuNP probe or AuNF probe sprayed on the conjugated pad, while Flu-OVA and goat anti-mouse IgG coated on the NC membrance. To make the best performance of AuNP/AuNF-LFIS, three important parameters including the optimal concentrations of goat anti-mouse IgG and Flu-OVA and amounts of AuNP/AuNF probes were determined. After a series of optimization experiments, the optimal conditions were determined as follows: the conditions of AuNP/AuNF-LFIS were 0.4 mg/mL of goat anti-mouse IgG on the C lines, 75 μg/mL/100 μg/mL of Flu-OVA on the T lines, 1.5 μL AuNP probe/1.75 μL AuNF probes on the conjugate pads.

**Fig. 4:**
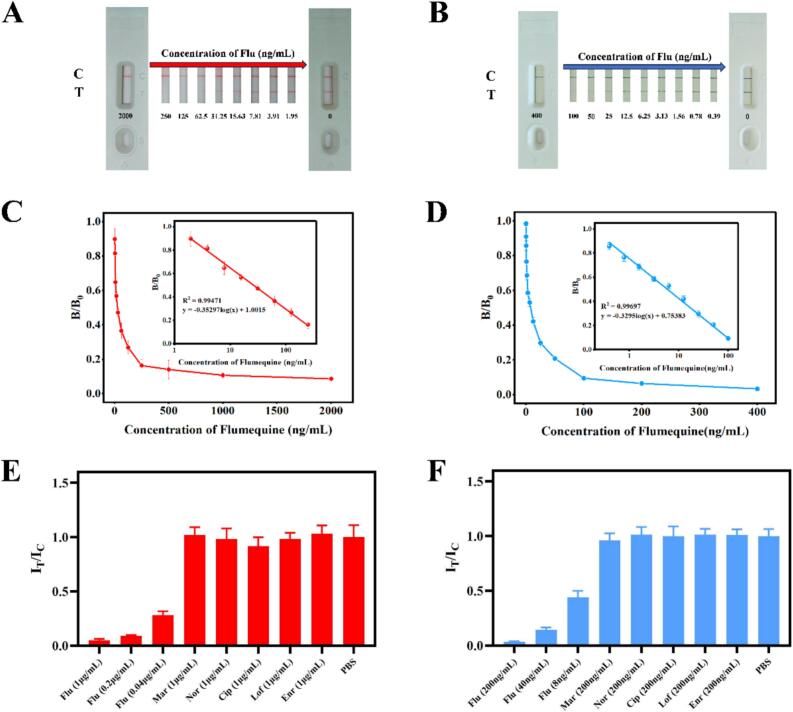
Performance evaluation of AuNP-LFIS and AuNF-LFIS. (A) Photo images of AuNP-LFIS detection with different concentrations of Flu. (B) Photo images of AuNF-LFIS detection with different concentrations of Flu. (C) Linear range and competitive curve of AuNP-LFIS for detecting Flu. (D) Linear range and competitive curve of AuNF-LFIS for detecting Flu. (E) Specificity of AuNP-LFIS. (F) Specificity of AuNF-LFIS.

The sensitivity of AuNP/AuNF-LFIS was evaluated with different concentrations (0–2000 ng/mL/0–400 ng/mL) of Flu standard solution after assembling LFIS under optimal conditions. The obtained competitive curves had great linear correlations in the range of 1.95–250 ng/mL and 0.39–100 ng/mL concentrations of Flu respectively. The calibration regression equations were y = −0.35297log(x) + 1.0015 (R^2^ = 0.99471) and y = −0.3295log(x) + 0.75383 (R^2^ = 0.99697), respectively. The vLOD of AuNP-LFIS and AuNF-LFIS were 3.91 ng/mL and 1.56 ng/mL respectively (Fig. 4A–B). Additionally, the LOD were 1.95 ng/mL and 0.39 ng/mL respectively (Fig. 4C–D). These results manifested that under the premise of good specificity, the sensitivity of AuNF-LFIS was 5 times and 2.5 times that of AuNP-LFIS under instrumental and naked eye observation, respectively. This improvement was due to stronger optical extinction effect and higher antibody binding efficiency of AuNF (Su et al., 2016). Furthermore, we have assessed the specificity of the proposed LFIS by examining groups of Flu at high, medium, and low concentrations to more accurately reflect real-world cross-reactivity risks. As shown in Fig. 4E–F, the specificity analysis demonstrated no significant change in I_T_/I_C_ values of fluoroquinolone analogues except Flu compared to negative control (PBS), indicating high selectivity of the developed AuNP-LFIS and AuNF-LFIS.

### 3.5 Detection of actual and spiked samples with LFIS

**Fig. 5:**
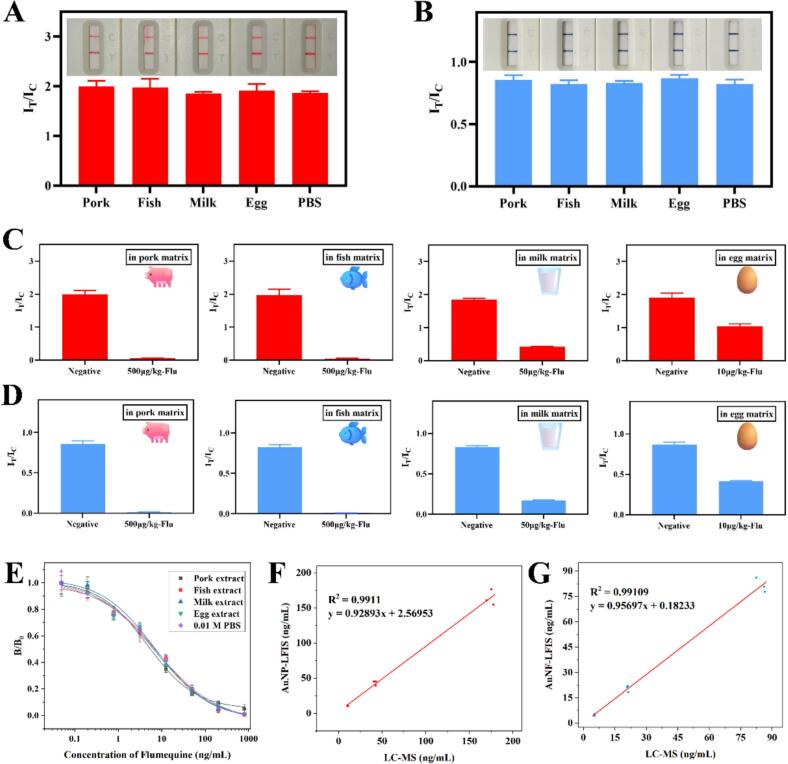
Actual sample test with LFIS and verification using LC-MS. (A) Actual sample detection with AuNP-LFIS. (B) Actual sample detection with AuNF-LFIS. (C) Comparison of detecting blank samples and spiked samples using AuNP-LFIS. (D) Comparison of detecting blank samples and spiked samples using AuNF-LFIS. (E) Matrix effects were evaluated by spiking kown concentrations of Flu into diverse food samples (pork, fish, milk and egg). (F) Consistency analysis of AuNP-LFIS and LC-MS detection results. (G) Consistency analysis of AuNF-LFIS and LC-MS detection results.

The recovery and variation coefficient (CV) of LFIS were shown in Table 1. In summary, the recoveries ranged from 90.35 % to 110.15 %, and CV ranged from 1.89 % to 6.91 %. The The variability of CV values maybe influenced by the type of sample and the input concentration of the Flu. Nevertheless, in numerous studies, a variation coefficient of approximately 10 % or less was deemed an acceptable detection results for immunochromatography (Ling et al., 2022; Sun et al., 2022). As shown in Fig. 5A–B, after extracting food samples of animal origin, AuNP-LFIS and AuNF-LFIS were respectively used to detect the extracts under the premise of 0.01 M PBS as negative control. There was no significant difference between I_T_/I_C_ values of actual samples and the I_T_/I_C_ value of PBS. Then, according to the MRLs in Chinese national standard GB31650-2019 and GB-31650.1-2022, the original extracts and the extracts spiked with Flu were detected by LFIS. The results showed that LFIS were also very sensitive to Flu in actual samples (Fig. 5C–D). Prior to spiking, it was confirmed that matrixes in the sample extracts had little effect on LFIS (Fig. 5E). In addition, LC-MS was explored to further evaluate the accuracy of the developed LFIS. From the detection results in Fig. 5F–G, it could be found that there was a good consistency between LC-MS and LFIS, indicating that the proposed LFIS were reliable in detecting Flu. Compared to the liquid chromatography with the limitations of long analysis time, complex pre-processing, and costly equipment (Gong et al., 2023), the proposed LFIS has advantages of high sensitivity, low-cost and simplified operational procedure were considered to be a powerful tool for the rapid and sensitive monitoring of Flu in real samples.

**Table 1:** Detection of Flu in spiked samples using AuNP-LFIS and AuNF-LFIS.

Method	Sample	Spiked (μg/kg)	Measured (μg/kg)	Recovery (%)	CV (%)
AuNP-LFIS	Fish	10	10.75 ± 0.48	107.47	4.51
		40	44.06 ± 1.66	110.15	3.76
		160	163.92 ± 11.32	102.45	6.91
	Milk	10	10.42 ± 0.67	104.25	6.44
		40	42.42 ± 2.83	106.04	6.68
		160	148.19 ± 2.80	92.62	1.89
AuNF-LFIS	Fish	5	4.52 ± 0.24	90.35	5.37
		20	20.49 ± 0.88	102.43	4.30
		80	75.63 ± 1.93	94.53	2.55
	Milk	5	4.89 ± 0.16	97.72	3.24
		20	19.05 ± 1.16	95.27	6.09
		80	77.69 ± 2.98	97.11	3.84

## 4 Conclusion

In conclusion, two types of highly sensitive and specific LFIS for monitoring Flu residues in foods of animal origin were developed. At first, we immunized mice with a complete antigen conjugated by carrier protein with Flu small molecule hapten, then these spleen cells were used for cell fusion. After several rounds of screening, hybridoma cell line 3B4 was obtained, which could steadily secrete high-affinity anti-Flu mAbs with good specificity. Based on anti-Flu mAbs, LFIS using AuNP and AuNF as signal amplification probes were developed, which could detect the presence of Flu in the sample solution within 10 min. The LOD and vLOD of the AuNP-LFIS were determined to be 1.95 ng/mL and 3.91 ng/mL, respectively. Compared to the AuNP-LFIS, the AuNF-LFIS exhibited more sensitive, achieving 0.39 ng/mL of LOD and 1.56 ng/mL of vLOD. Considering the cost-effectiveness and sensitivity of the proposed LFIS, it is our belief that the LFIS developed in the study could serve as a powerful tool to detect Flu in the area of food safety, environmental monitoring and public health. Although the excellent performance of the LFIS, the storage stability also needed to be further investigated to ensure their practical applicability.

## CRediT authorship contribution statement

**Yuxuan Liu:** Writing – original draft, Methodology, Formal analysis, Data curation, Conceptualization. **Kang Jiang:** Validation, Methodology, Formal analysis, Data curation. **Xiaoli Li:** Validation, Formal analysis, Data curation. **Linfang Lu:** Visualization, Validation, Data curation. **Menghan Sun:** Validation, Methodology. **Na Li:** Validation, Formal analysis. **Lakshani Madushika:** Validation, Formal analysis. **Jun Yuan:** Writing – review & editing. **Sumei Ling:** Writing – review & editing, Methodology. **Shihua Wang:** Writing – review & editing, Supervision, Conceptualization.

## Ethical statement

All parts of the experiments involving animals were carried out according to EU Directive 2010/63 for the protection of animals and approved by the Ethics Committee (license number: PZCASFAFU24118) of the Fujian Agriculture and Forestry University of China.

## Declaration of competing interest

The authors declare that they have no known competing financial interests or personal relationships that could have appeared to influence the work reported in this paper.

## Data Availability

Data will be made available on request.
